# The macro-evolutionary events in esophageal squamous cell carcinoma

**DOI:** 10.18632/oncotarget.22625

**Published:** 2017-11-15

**Authors:** Bin Yang, Ting Yan, Heyang Cui, Enwei Xu, Yanchun Ma, Caixia Cheng, Ling Zhang, Pengzhou Kong, Fang Wang, Yu Qian, Jian Yang, Yaoping Li, Hongyi Li, Yanghui Bi, Xiaoling Hu, Juan Wang, Bin Song, Jie Yang, Wei Gao, Jing Liu, Binbin Zou, Ruyi Shi, Yanyan Zhang, Haiyan Liu, Yiqian Liu, Yuanfang Zhai, Lu Chang, Yi Wang, Yingchun Zhang, Zhiwu Jia, Xing Chen, Yanfeng Xi, Guodong Li, Jianfang Liang, Jiansheng Guo, Shiping Guo, Rongsheng Zhang, Xiaolong Cheng, Yongping Cui

**Affiliations:** ^1^ Translational Medicine Research Center, Shanxi Medical University, Taiyuan, Shanxi, China; ^2^ Shanxi Key Laboratory of Carcinogenesis and Translational Research on Esophageal Cancer, Shanxi Medical University, Taiyuan, Shanxi, China; ^3^ Key Laboratory of Cellular Physiology, Ministry of Education, Shanxi Medical University, Taiyuan, Shanxi, China; ^4^ Department of Tumor Surgery, Shanxi Cancer Hospital, Taiyuan, Shanxi, China; ^5^ Department of Pathology, Shanxi Cancer Hospital, Taiyuan, Shanxi, China; ^6^ Department of Pathology, The First Hospital, Shanxi Medical University, Taiyuan, Shanxi, China; ^7^ Department of Colorectal & Anal Surgery, Shanxi Provincial People's Hospital, Taiyuan, Shanxi, China; ^8^ Department of General Surgery, The First Hospital, Shanxi Medical University, Taiyuan, Shanxi, China; ^9^ Department of Endoscopy, Shanxi Cancer Hospital, Taiyuan, Shanxi, China

**Keywords:** esophageal squamous cell carcinoma (ESCC), whole genome doubling (WGD), neutral loss of heterozygosity (NLOH), telomere-bounded copy number alterations (TCNAs), genome evolution

## Abstract

Understanding the evolutionary processes operative in cancer genome may provide insights into clinical outcome and drug-resistance. However, studies focus on genomic signatures, especially for macro-evolutionary events, in esophageal squamous cell carcinoma (ESCC) are limited. Here, we integrated published genomic sequencing data to investigate underlying evolutionary characteristics in ESCC. We found most of ESCC genomes were polyploidy with high genomic instability. Whole genome doubling that acts as one of mechanisms for polyploidy was predicted as a late event in the majority of ESCC genome. Moreover, loss of heterozygosity events were more likely to occur in chromosomes harboring tumor suppressor genes in ESCC. The 40% of neutral loss of heterozygosity events was not a result of genome doubling, suggesting an alternative mechanism for neutral loss of heterozygosity formation. Importantly, deconstruction of copy number alterations extending to telomere revealed that telomere-bounded copy number alterations play a critical role for amplification/deletion of oncogenes/suppressor genes. For well-known genes *SOX2*, *PIK3CA* and *TERT*, nearly all of their amplifications were telomere bounded, which was further confirmed in a Japanese ESCC cohort. Furthermore, we provide evidence that karyotype evolution was mostly punctuated in ESCC. Collectively, our data reveal the potential biological role of whole genome doubling, neutral loss of heterozygosity and telomere-bounded copy number alterations, and highlight mecro-evolution in ESCC tumorigenesis.

## INTRODUCTION

Genomic studies have revealed an extensive genetic heterogeneity generated through genomic instability not only between different tumors but also intra-tumor [1]. Chromosomal instability (CIN) represents a dynamic state that likely to contribute to intra-tumor heterogeneity by creating a genetically distinct pool of tumor cells. Of the tumor cell pool, beneficial mutations conferring a selective advantage on the cell may arise, followed by a successive wave of clonal expansion [2–4]. CIN has profound effects on the cell genome and is a common trait of more than 70% of human cancers, therefore is implicated as an initiator of tumorigenesis [3]. In addition, CIN may facilitate the adaptation of tumors to environmental or stromal stress and is implicated in determining tumor progression and associated with poor outcome, tumor relapse, and multi-drug resistance across a range of cancer types [5]. Exploiting tumor CIN status and defining how it generate genetic diversity and shape genome evolution may provide insights into clinical outcome, treatment failure and assist prognostic predictions and therapeutic target. Unfortunately, the CIN status and how it shapes genome evolution in ESCC have not been fully understood.

Recent literatures report the contribution of whole-genome doubling (WGD) to CIN and tumor evolution [6]. WGD event was inferred to occur both before and after other copy-number alterations (CNAs) across various cancer types [6]. Moreover, the types of losses in a genome-doubled sample shed light on the timing of genome doubling (GD) relative to copy-number losses in the genome [7]. Copy number losses that occur on the background of a diploid genome before GD will result in loss of heterozygosity (LOH) whereby one of the parental alleles is lost [8]. The contribution of GD to CIN and how GD could affect genome evolution in human cancer has been investigated, for example, a recent study demonstrates that the tolerance of GD propagates and accelerates genome evolution in colon cancer [9]. Previously, we report nearly 70% of ESCC genomes suffer GD events [10]. Hence, it is of interest to elucidate the genomic differences between tumors with or without GD, and assess the impact of GD on the evolution of ESCC.

Previously, we have reported the role of APOBEC family of cytidinedeamiases in mutagenesis and identified its connection with hotspot mutations of *PIK3CA* in ESCC [11]. In addition, a Japanese ESCC study displays an association of APOBEC signature with *ZNF750* mutations [12]. Assuredly, the signatures of genomic instability could be extended to genomic aberrations, for example, allelic imbalance at telomere is a marker for deficient homologous recombination repair, and it is also predictive of benefit from DNA damaging in breast and ovarian cancers [13]. In ESCC, through whole-genome analyses, we observed diverse models of genomic signatures including breakage-fusion-bridge (BFB), chromothripsis and kategsis, which frequently lead to oncogene amplifications such as *CCND1* and *FGFR1* [10]. Recent studies report that the cause of these genomic signatures may be attributed to telomere dysfunctions [14, 15]. These findings highlight the importance of telomere-bounded CNAs (TCNAs) in ESCC tumorigenesis. Given the complexity of cancer genome that consists of genomic changes from point mutation to larger-scale copy number alteration or WGD, characterization of the potential genomic signatures and their mutational ordering may provide useful insights into the ESCC genome evolution.

In this study, we combined the sequencing data of our previous cohorts to explore the potential genomic signatures and the impact of GD on evolution in ESCC [10, 11, 16]. Our data reveal frequent genomic signatures of NLOH not derived from GD and TCNAs that cause amplification of cancer-associated genes in ESCC. We also provide evidence that karyotype evolution was punctuated in most of ESCCs.

## RESULTS

### High genome instability of ESCC genomes

**Figure 1:**
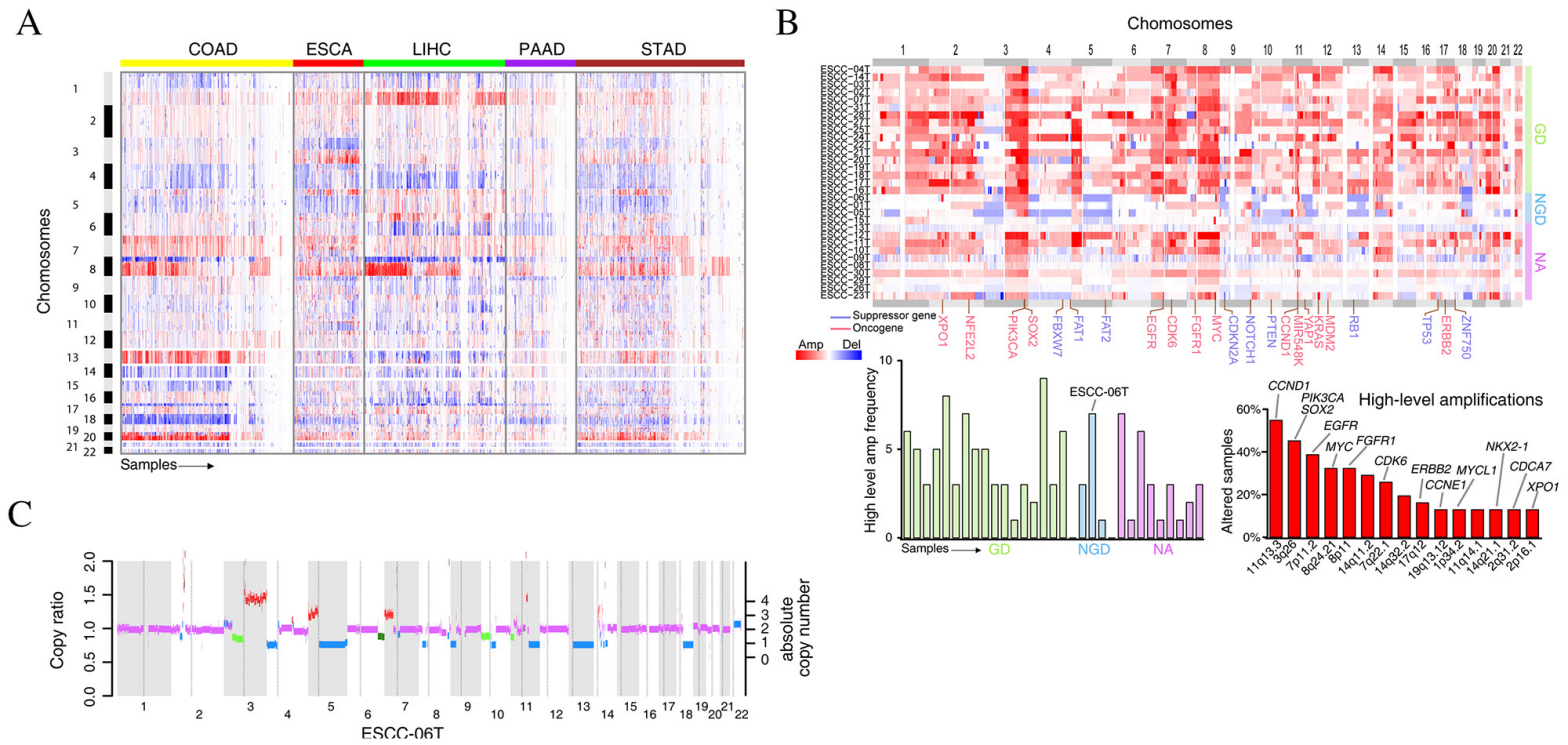
The genome instability in esophageal cancers **(A)** The copy number profiles across five types of gastrointestinal tumors from TCGA datasets. Tumors are plotted by horizontal axis, chromosome positions are arranged vertically. **(B)** The genome instability of 31 ESCC tumors. The upper panel shows the SCNAs while the bottom panels show the high-level amplification frequency of each tumor (left panel) and gene (right panel). **(C)** Copy number variations for ESCC-06T. Green segments represent the subclonal deletion.

To assess structural and numerical CIN and provide insight into genomic instability across cancers, we integrated copy number profiles of 1660 cancer specimens from 5 types of gastrointestinal tumors from the Cancer Genome Atlas (TCGA) [17, 18]. Four types of tumors including colorectal carcinoma (COAD), liver hepatocellular carcinoma (LIHC), pancreatic adenocarcinoma (PAAD) and stomach adenocarcinoma (STAD) were found to be divided into two classes: one class with high somatic copy number alteration (SCNA) shows copy number changes converging to specific chromosomes such as chr8 amplification and 4q deletion; the other class with low SCNAs shows few copy number alterations and may evenly be chromosomal stable. Conversely, largely copy number alterations (CNAs) were observed in almost all of esophageal carcinoma (ESCA) and frequent copy-number changes were not clustered in specific chromosome (Figure 1A). To further investigate the genome instability of ESCC, we analyzed CNAs from whole-genome sequencing (WGS) data of 31 ESCCs. Strikingly, we found that 17 out of 31 ESCC genomes had occurred WGD events (Figure 1B, upper panel) and 24 of ESCC genomes harbored more amplifications than deletions (Supplementary Figure 1A). Most known oncogenes (e.g. *EGFR, FGFR1, MYC, ERBB2*) or tumor suppressors (e.g. *CDKN2A, NOTCH1*) were detected to reside within the focal SCNA regions. Furthermore, high-level amplification of regions harboring oncogenes (such as *CCND1* (17/31), *SOX2* (14/31), *EGFR* (12/31), *MYC* (10/31), *FGFR1* (10/31)) have consistently been observed in 29 out of 31 ESCCs, except for 2 genomes that had not undergone GD (Figure 1B, bottom panel). It is worth noting that, instead of missense mutations found in *XPO1* [19], recurrent focal amplification of *XPO1* was identified in 5 out of 31 ESCCs (Supplementary Figure 1B). Together with the protein over-expression of XPO1 revealed by De-Chen Lin *et al* [19], these data indicate *XPO1* may act as a therapeutic target in ESCC.

By using ABSOLUTE, we could distinguish clonal and subclonal somatic single-nucleotide variations (SNVs) in ESCC. We found that majority of ESCCs were genetically heterogeneous harboring subclonal populations of cells. Specially, a non-genome doubling (NGD) ESCC-06T with poor prognosis that has much more high-level amplification peaks exhibited high intra-tumor heterogeneity. Approximately 33% of the somatic mutations of this patient were subclonal mutations (Supplementary Figure 1C). Interestingly, we also observed subclonal deletion of multiple chromosomes, including 10p, partially of 3p, 11p in approximately 70% of tumor cells and partially of 6p in approximately 60% of tumor cells in this patient (Figure 1C). Together, our results suggest the high genome instability and its important evolutionary role in ESCC.

### Genome doubling events and its impact on evolution in ESCC

**Figure 2:**
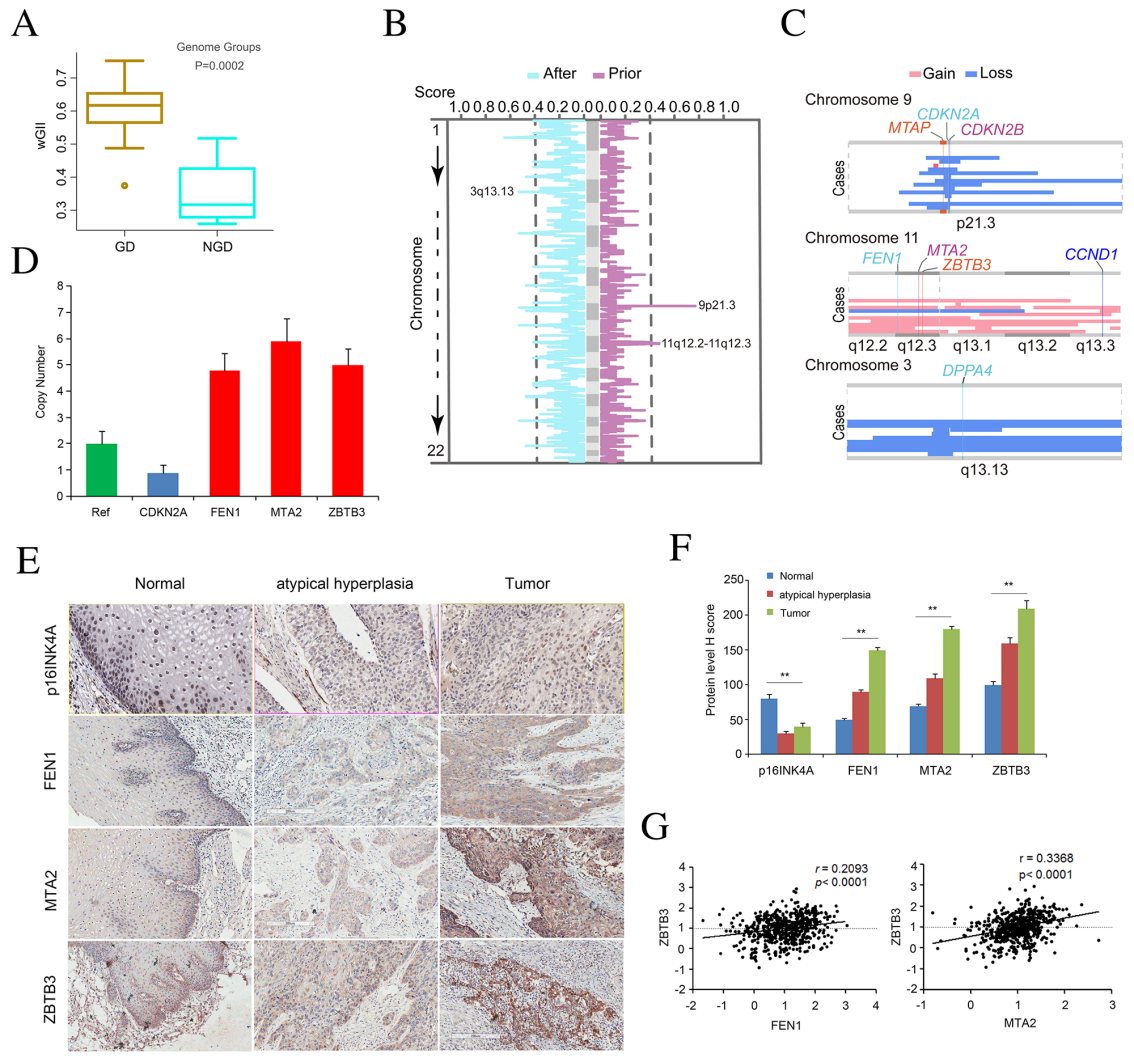
Copy number alteration for peak regions that were considered as early events in ESCC **(A)** Boxplot of the weighted Genome Instability Index (wGII) in individuals with or without GD. One-way ANOVA test was used to compare the differences between the two groups. **(B)** The copy number altering score for event set prior to GD and event set after GD are shown in the panel. Horizontal axis represents the copy number altering score. The two curves are on behalf of the percentage of samples with copy number variation in the gene region across the event set. A peak at one curve means that the region with some genes has variated frequently. **(C)** The state of three selected peaks with some genes for three chromosomes. In each plate, vertical represents cases with GD. **(D)** Copy number of *CDKN2A, FEN1, MTA2, ZBTB3* assayed by qPCR in tissue-microarray containing 36 atypical hyperplasia tissues and 72 of ESCC tumors. The RNase P gene was used as reference normal (red). Data are mean ± SD. All assays were performed in triplicate. **(E)** Represent images display immunohistochemical staining for p16INK4A, FEN1, MTA2, and ZBTB3 in atypical hyperplasia tissues and ESCC tumors. **(F)** Quantification of expression level of p16INK4A, FEN1, MTA2, and ZBTB3 in atypical hyperplasia tissues and ESCC tumors compared to that of normal esophagus tissue based on judgment of IHC staining intensity. **(G)** Correlation analysis of protein level of FEN1, MTA2, and ZBTB3.

In our cohort, 17 of GD cases and 4 of NGD cases were distinguished with the copy number profiles. To assess whether GD events could enhance genomic instability, the modified Genome Instability Index (wGII) was used [20]. Interestingly, significantly higher wGII was observed in tumors with GD events compared to those that had not undergone GD (Figure 2A, one way ANOVA test, *P* = 0.0002). Compared to the NGD tumors, GD tumors have large amounts of focal SCNAs (Supplementary Figure 2A, 234 per GD case *versus* 142 per NGD case). These results suggest a potential relationship between GD events and genome complexity in ESCC.

To further explore the genomic differences before and after GD, we classified focal events into early or late events and determined the temporal relations of individual SCNAs to GD using different approaches [21]. In GD tumors, we found most focal SCNAs occurred after GD events (Supplementary Figure 2A), indicating that GD was inferred to occur earlier relative to focal SCNAs in these tumors. To nominate gene aberrations in early or late stage of ESCC, we then estimated copy number altering score for each gene. Interestingly, we found two peak regions that contained deletion of 9p21.3 and amplification of 11q12.2-11q12.3 were considered as early events (Figure 2B). These two peaks are most likely to contain oncogenes or Tumor Suppressor Genes (TSGs). Region 9p21.3 contained three genes (*CDKN2A*, *CDKN2B*, *MTAP*) known to be inactivated by homozygous deletion (Figure 2C). Importantly, copy-number analyses verified the deletion of *CDKN2A* and immunohischemistry (IHC) staining in tissue-microarray containing 36 atypical hyperplasia tissues and 72 of ESCC tumors confirmed the loss of expression of p16INK4A (Figure 2D-2F), indicating that *CDKN2A* depletion may be a potential biomarker for early detection of ESCC. In addition, region 11q12.2-12.3 contained *FEN1*, *MTA2* and *ZBTB3* (Figure 2C). In parallel, we verified the amplifications of these candidate genes and the over-expression of these proteins in 36 of atypical hyperplasia tissues and 72 of ESCC tumors (Figure 2D-2F). Specially, contrary to FEN1's role as a tumor suppressor in some types of cancer [22], our chi-square test suggests that the over-expression of FEN1 in atypical hyperplasia and ESCC tumor tissues was statistically significant (*P* < 0.001), account for 53% of ESCCs (Figure 2F), indicating that *FEN1* may be required to support the growth and progression of ESCC. Notably, we found the statistic positive correlation of FEN1 expression with ZBTB3 (*r* = 0.2093, *P* < 0.0001) while ZBTB3 expression was positively associated with MTA2 expression (*r* = 0.3368, *P* < 0.0001) (Figure 2G), indicating that these genes may be oncogenes and potential drug targets in ESCC. Simultaneously, cancer stemness gene *DPPA4* that located at 3q13.13 and was selected in the set of events after GD (Figure 2C) may contribute to the development and progression of ESCC. The biological function of these alterations in the initiation and progression of ESCC needs further investigation.

**Figure 3:**
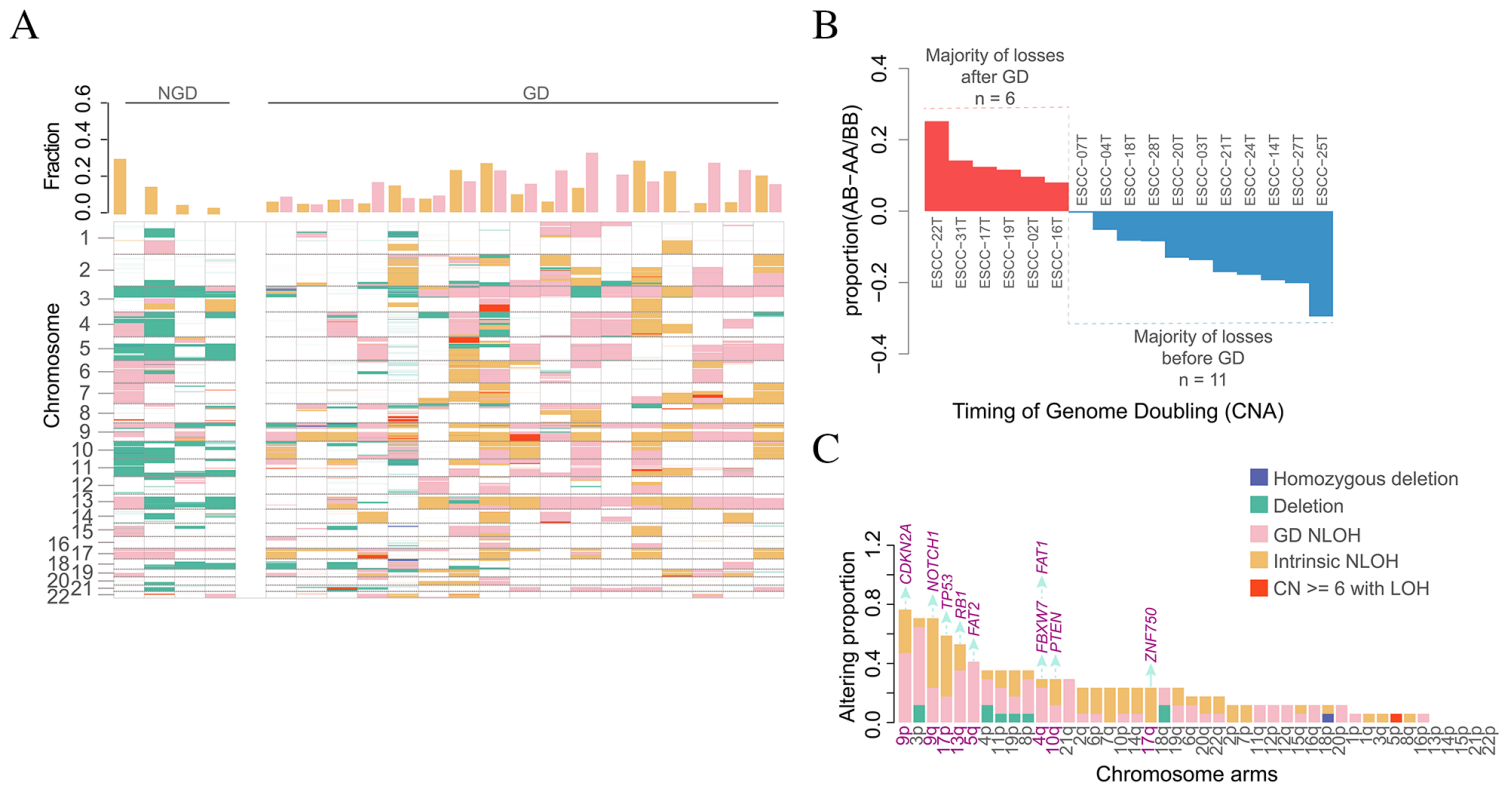
The impact of genome doubling on evolution **(A)** LOH states of early tumor with NGD and GD. Different colors represent different type of LOH event. The bar plot in upper panel depicts the genome fraction that displays GD-derived NLOH and Intrinsic NLOH for each case. The details of LOH events for each ESCC are shown in the bottom panel. **(B)** The timing of GD estimated using copy number and neutral LOH profiles. Each bar represents one GD case and its height is the difference using the proportion of AB - proportion of AA/BB. Tumor genomes with majority of losses after GD are shown in red (n=6; proportion AB > proportion AA/BB), whereas the others are shown in blue (n=11; proportion AB < proportion AA/BB). **(C)** The altering proportions of tumor with arm-level LOH events (segment length > 0.75 chromosome arm length). Each bar represents one chromosome arm and different colors represent different LOH event. Horizontal axis represents those arms Listed from largest to smallest with proportion. Vertical axis represents the genome fraction. Tumor suppressor genes (purple) are shown on top of the bar of those chromosome arms (purple).

Additionally, we characterized LOH events across 21 ESCC genomes and found NLOH was very prevalent in both GD and NGD ESCCs (Figure 3A). Moreover, GD was a relatively late event in the majority of ESCCs (Figure 3B). In GD tumors, we have distinguished approximately 40% NLOH events not as a result of GD and these NLOH events were defined as intrinsic NLOH. There was an average of 60 intrinsic NLOH events per case for 17 of GD tumors, suggesting that, besides GD, alternative mechanism may contribute to NLOH in ESCC. Furthermore, we observed that NLOH frequently occurred on several chromosomes in ESCC. It is particularly strong that heterozygosity of chromosome 9p and 17p was loss in 16 out of 17 GD tumors (94%) and in 11 out of 17 GD tumors (65%), as well as chromosome 5q (47%), 9q (88%), 13q (76%), 3p (82%), respectively, suggesting that deletion on chromosomes 3p, 5q, 9p, 9q, 13q and 17p occurred before GD events during the development of ESCC. Notably, as a result of NLOH occurred at *TP53* (65% of GD cases), *RB1* (71% of GD cases), *NOTCH1* (82% of GD cases), *FBXW7* (35% of GD cases), *FAT1* (30% of GD cases), *FAT2* (47% of GD cases), *CDKN2A* (18% of GD cases) and *PTEN* (42% of GD cases), NLOH events are more likely to occur in chromosomes harboring TSGs (Figure 3C, Supplementary Figure 2B; Wilcoxon rank sum test, *P* = 9.308e-05). These results suggest that an abundance of combinations of deletions involving suppressor genes may play an important role in the tumorigenesis of ESCC.

Finally, to investigate the impact of GD on dynamics of mutational spectrum, we analyzed the types of somatic SNVs before and after GD. In total, we detected 257,430 somatic SNVs across the 17 GD samples, of which 18,069 were identified as subclonal mutations by ABSOLUTE. Although more somatic SNVs occurred after GD, mutational spectrum proportions were similar for before and after GD (Supplementary Figure 3A, Wilcoxon rank sum test, *P* = 0.7671), which was further confirmed by 96 of GD tumors of ESCC with whole exome sequencing data available (Supplementary Figure 3B, Wilcoxon rank sum test, *P* = 0.57), suggesting that mutational spectrum was not affected by GD events. We further compared APOBEC mutational signature between clonal and subclonal mutations and found no significantly variations (Supplementary Figure 3C-3D). Thus, unlike non-small cell lung cancer (NSCLC) and bladder cancer [23], APOBEC signature plays a key role in generating both clonal and subclonal mutations and may contribute to both initiation and progression of ESCC.

### Telomere-bounded copy number alterations (TCNAs)

**Figure 4:**
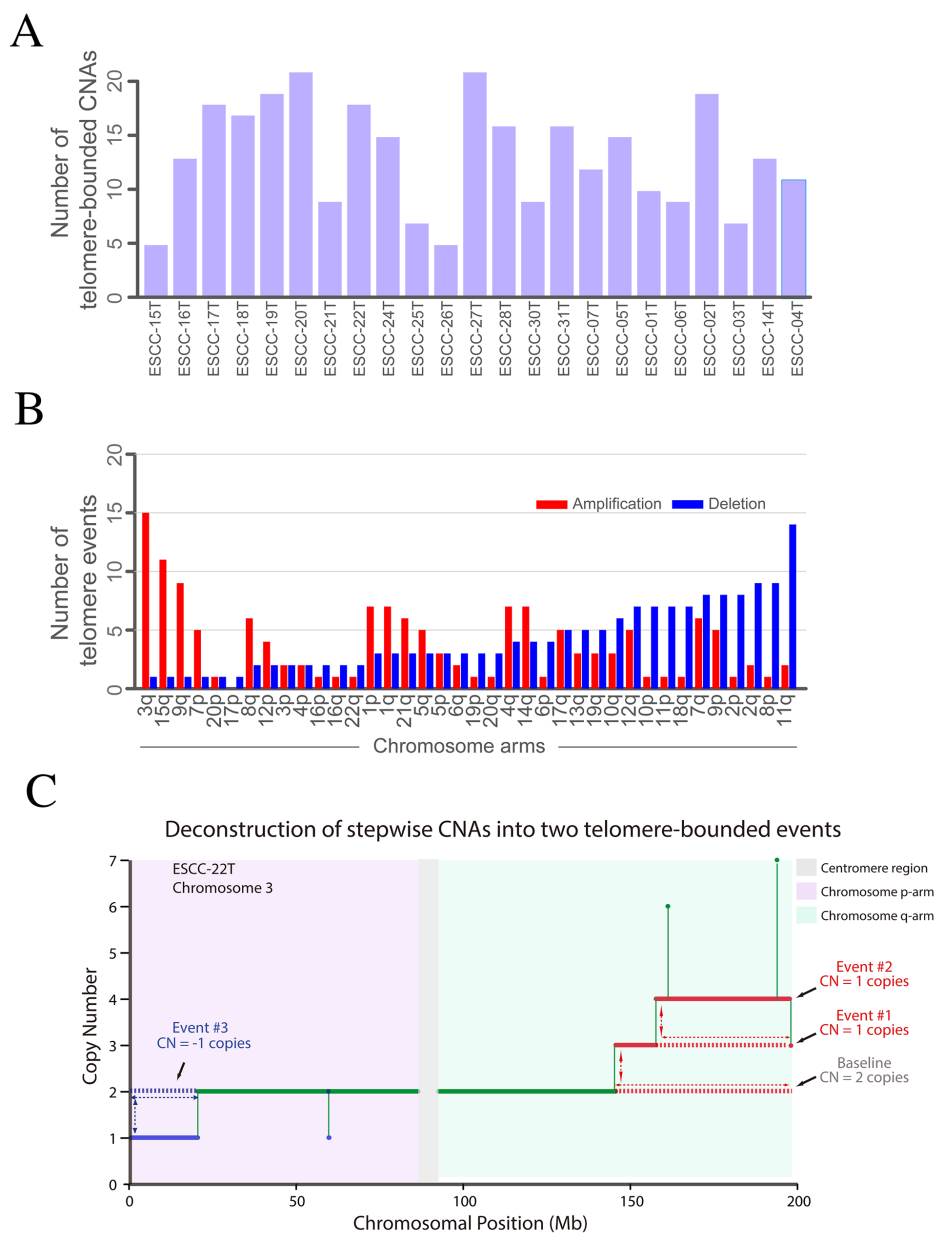
Telomere-bounded copy number changes and targeted amplification across 23 ESCCs **(A)** The distribution of TCNAs in each tumor. **(B)** The telomere-bounded amplification or deletion for each chromosomal arm. **(C)** Example of deconstruction of stepwise CNAs in chromosome arm into underlying telomere-bounded copy number alterations. Green line represents copy number baseline with 2 copies, red lines represent two underlying telomere-bounded amplifications, and in contrast, blue line represents telomere-bounded deletion.

To explore the genetic feature of TCNAs and its affected genes in ESCC, we used centrosome as reference to define telomere-bounded amplification or deletion when absolute copy number of telomere-bound segments was more or less than centrosome respectively. Totally, we found an average of TCNAs per tumor ranging from 5 to 20 (Figure 4A). The number of telomere-bounded deletion was comparable to amplification, indicating the balance of telomere gain and loss in ESCC genome. The most frequent telomere-bounded amplification occurs in chromosome 3q and 15q that have fewer telomere-bounded deletions (Figure 4B). Similarly, the telomere-bounded deletion of 11q shows little telomere-bounded amplification. These findings suggest these TCNAs in chromosomes were positively selected in ESCC genomes.

Telomere-bounded deletion is a key step for BFB formation and BFB-derived amplification of oncogenes such as *CCND1*, *EGFR* and *FGFR1* were observed (Supplementary Figure 4A). In our data, 40% of 11q telomere-bounded deletion accompanied with BFB cycles. Of note, telomere-bounded deletion of 11q not only leads to the BFB cycles that cause high-level amplification of *CCND1* but also deletion of TSGs (e.g. *FAT3*) (Supplementary Figure 4B), suggesting the dual role of telomere-bounded deletion in tumorigenesis of ESCC. Meanwhile, 15 out of 21 have telomere-bounded amplification in chromosome 3q and most of 3q amplifications could be deconstructed into several underlying telomere-bounded amplifications (Figure 4C). Consistent with previous report, the high-level peak located in 3q26-3q telomere that harboring several well-known cancer genes, such as *SOX2* and *PIK3CA*. In our data, nearly all of *SOX2* amplifications were TCNA, which could be further validated in a Japanese ESCC cohort [12]. Of note, we also found amplification of *TERT* was telomere-bounded in ESCC. To further explore potential cancer-associated gene in 3q, we exclude TCNA of 3q and a focal amplification of 3q28 containing gene *TP63* emerged (Supplementary Figure 5A). In addition, we also found telomere-bounded amplification involving gene *IQGAP1* in chromosome 15 (Supplementary Figure 5B). Collectively, across ESCC genomes, we found that focal, telomere-bounded SCNAs accounted for more SCNAs than expected assuming random SCNA locations. These results highlight the critical role underlying mechanism of internal CNAs and the positively selected TCNAs in ESCC genomes.

### Evolution of ESCC genome

**Figure 5:**
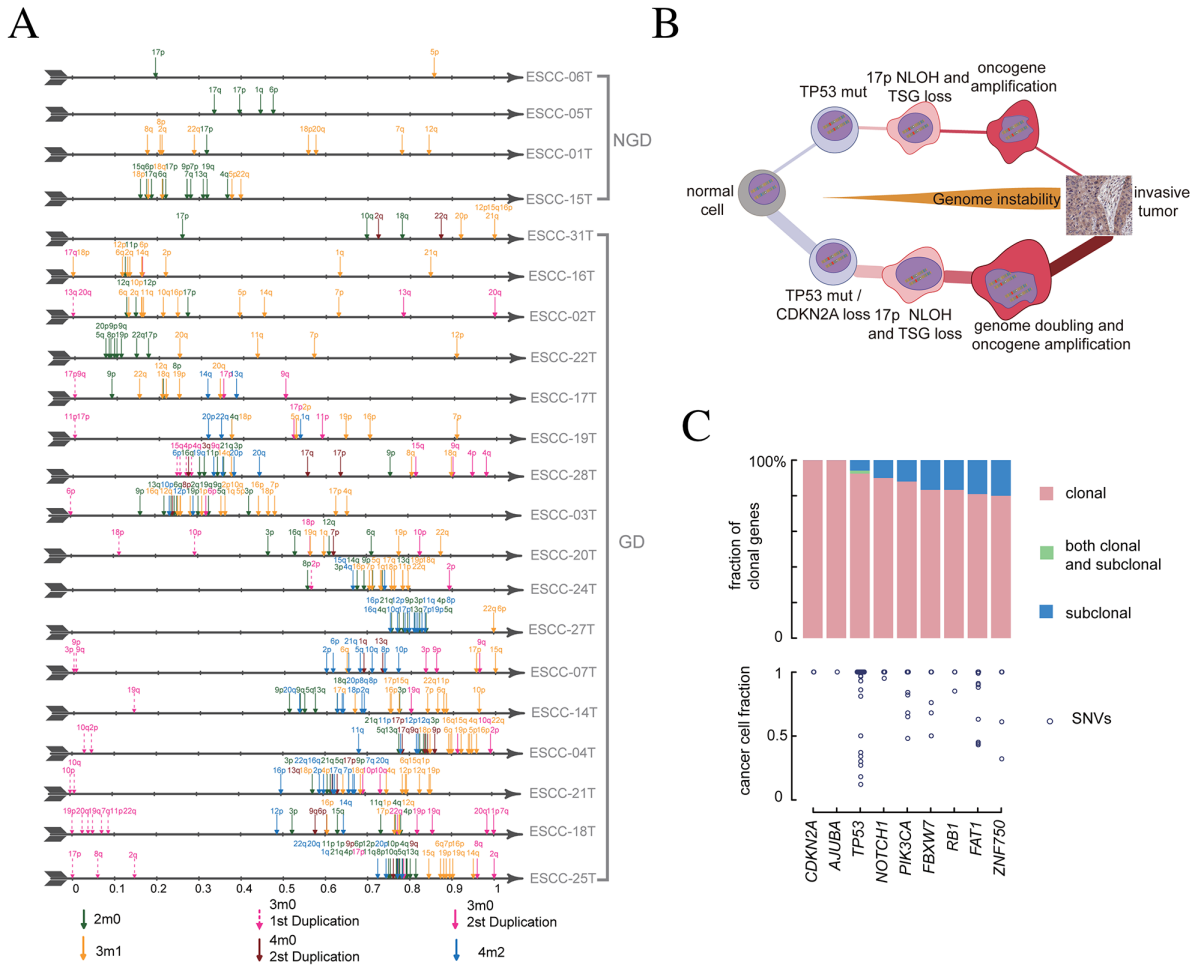
Molecular time and evolutionary path of ESCC **(A)** Molecular time of chromosomal gain events. Molecular time point is shown as an arrow with different colors, each represents a type of amplification events. **(B)** The two general routines to develop ESCC. The top model represents a non-genome-double way, characterized with *TP53* inactivation and large number of LOH events. The bottom model represents a genome-doubled pattern involving early inactivation of *CDKN2A/TP53* followed by formation of neutral LOH, followed by genome doubling. The genomic instability begins early and works during the whole evolution process. **(C)** The clonal status and cancer cell fraction of driver genes in 238 ESCCs. On the top, driver genes are classified as clonal or subclonal and CCF value of each SNVs are drawn at bottom.

21 ESCC genomes with clearly absolute copy number were used to infer the chromosomal molecular time (Supplementary Table 1). We found that chromosomal gains in 11 out of 21 individuals occur at less than 10% of point mutation time (Figure 5A), indicating that chromosomal instability begins early in ESCC evolution. Strikingly, we observed that arm-level changes were more likely to enrich and occur in a short period of time instead of continual gain or less in uniform space, suggesting that karyotype evolution was punctuated in most of ESCC genomes.

To further characterize the consensus path that shapes ESCC genomes, we analyzed the molecular time of chromosomal gain events and driver mutations, and summarized three key steps in ESCC tumorigenesis. First, the homozygous deletion of *CDKN2A* and *TP53* mutations were the most common alterations in ESCC cohort regardless of GD events. There were 15 ESCCs having homozygous *CDKN2A* (9p21.3) deletion and 5 tumors harboring NLOH or deletion of chr9p (Supplementary Figure 6A, left panel). In the 15 tumors with *TP53* mutations, 11 tumors have mutations at least two alleles, which suggest that *TP53* mutation precedes its amplification (Supplementary Figure 6A, right panel). High proportion of homozygous *CDKN2A* loss and *TP53* mutation also support that both of them tend to occur before the amplification. Next, we compared the molecular time of intrinsic NLOH as it was very prevalent and associated with the distribution of TSGs in ESCC. We observed that the first round NLOH occurred early during the evolution process, especially in GD tumors, as molecular time of the first round of NLOH was less than 0.1 in most of ESCC genomes (Figure 5A). The most frequent arm of NLOH is chr17p, which account for more than half of samples (Supplementary Figure 6B), further supporting *TP53* mutations is an early event in most of ESCCs. Finally, we directly evaluated the molecular time of GD events in 11 of 17 individuals. Consistently, GD was a late event in these samples, occurring after more than 50% of molecular time (Supplementary Figure 6C). Altogether, these results implied a consensus path of ESCC evolution, beginning with the *CDKN2A/TP53* mutations, followed by NLOH, and ultimately, some of them suffer GD (Figure 5B and Supplementary Figure 6D).

Interestingly, we found that mutations of significantly mutated genes were almost clonal mutations in our 21 WGS samples (Supplementary Table 2). We further explored it in our whole exome sequencing data of 161 ESCC [11]. We synthetically identified 9 driver genes in 161 individuals and the frequency of clonal genes were almost larger than 0.8 (Figure 5C). The detection of rare sub-clonal driver genes in sequencing data may suggest a rapid clonal sweep process directed by these driver genes (Supplementary Figure 7A). Specially, we found the coexistence of clonal and subclonal mutations for *TP53* gene in case ESCC-315T and ESCC-260T. Moreover, we found that the multiplicity of oncogenes such as *PIK3CA* were generally smaller than its absolute copy number while the multiplicity of tumor suppressor genes tends to be equal to its absolute copy number (Supplementary Figure 7B), suggesting that oncogenes altered later than TSGs in ESCC.

## DISCUSSION

By systematically profiling copy number alterations and GD events, we identified high-level of genomic instability in ESCC, and most of them are more likely to follow punctuated evolution. Of note, we display diverse model of macro-evolutionary events operative in ESCC evolution, such as GD, NLOH and TCNAs; these genomic signatures may have important roles in ESCC tumorigenesis. Moreover, the high rate of NLOH suggests that epigenetic abnormalities of many particular genes might be involved in the development and progression of ESCC.

We and others previously reported various forms of genomic instability across ESCC genomes. WGD, observed in 70% of ESCC genomes, was found to be associated with higher rates of CIN and genome complexity in ESCC. A GD event could represent a macro-evolutionary leap in tumors and can drastically alter the evolution by, for example, activating oncogenes [6, 7]. Our result sheds light on the importance of WGD events in ESCC evolution. Unlike colorectal cancers in that GD is an early event [9, 18, 24], we found, in the majority of ESCC tumors, GD likely occurred as a relatively late event, after *CDKN2A/TP53* mutations and NLOH, suggesting that CIN occurs before GD in ESCC evolution. Moreover, our results support the high genome instability residing in ESCC and its important evolutionary role for tumor progression. Accordingly, stratifying patients’ responses according to CIN status should be considered in the design of clinical trials to test novel anticancer agents in ESCC and minimize the confounding effects of tumor CIN status on drug sensitivity.

It is well known that high-level amplification is a marked feature of CIN and always accompanies with over-expression of oncogenes leading to tumor progression [1–3]. Several genes on chromosomal region 11q12.2-12.3 that were amplified in our study are known oncogenes whose amplification has been associated with poor prognosis. For example, *FEN1* (Flap structure-specific Endonuclease 1) is required for DNA replication repair, epigenetic inheritance, and cell cycle control. Functional deficiency in *FEN1* has been shown to cause genomic instability, chronic inflammation and cancer; thus may be a potential candidate cancer susceptibility gene [22]. Previous studies based on GWAS approach have shown that *FEN1* was significantly associated with ESCC and head and neck squamous cell carcinoma (HNSCC) [25, 26]. *ZBTB3* is an essential factor for cancer cell growth via the regulation of the ROS detoxification pathway [27]. *MTA2* has been shown to deacetylate p53 and to repress p53-dependent transcriptional activation [28, 29]. We also observed copy number gain in *FEN1*, *MTA2* and *ZBTB3,* suggesting that copy number gain is responsible for overexpression of these genes in at least a subset of patients with ESCC. The involvement of *FEN1, ZBTB3,* and *MTA2* in controlling genomic stability suggests that functional dysregulation of these genes through mutations would facilitate further tumor mutagenesis, raising the possibility of these oncogenes as potential therapeutic targets for ESCC patients.

Recently, Jia-Jie Hao and colleagues report temporal clonal evolution from M-seq of 51 tumor regions from 13 ESCC cases [30]. In their study, half of the driver mutations located on the branches of tumor phylogenetic trees targeted oncogenes, including *PIK3CA*, *NFE2L2* and m*TOR* whereas the majority of truncal and clonal driver mutations occurred in tumor-suppressor genes, including *TP53*, *KMT2D* and *ZNF750*. However, the analysis based on multiregional whole-exome sequencing is not able to completely resolve the true temporal ordering of all somatic variants. In our study, the WGS analyses indicate that arm-level changes were enriched and occurred in a short period of time, supporting that karyotype evolution was punctuated in most of ESCC genomes. Other examples of punctuate evolutionary events occurring in ESCC have been previously proposed, such as chromothripsis involving complex chromosomal rearrangement events [10]. Moreover, several driver mutations that were found in all tumor cells among these 21 ESCC genomes can be placed on the shared trunk of the phylogenetic tree, including mutations of *TP53, CDKN2A, AJUBA, PIK3CA, NOTCH1, FBXW7, RB1, FAT1*, and *ZNF750*. Identifying trunk mutations can be helpful because targeting subclonal alterations would likely be less effective compared with therapies targeting true founding clone alterations [5, 7, 31, 32]. Therefore, targeting these trunk mutations may provide clinical application to develop therapeutic paradigms for ESCC patients. For example, *PIK3CA* showed recurrent activating mutations suggesting that this gene may be potential therapeutic target for ESCC patients harboring *PIK3CA* mutation. *NOTCH1*, *CDKN2A*, *FBXW7, FAT1*, and *ZNF750* were considered as tumor suppressors and showed recurrent inactivating mutations. Although these results possibly limited the potential value of targeted therapy, there might be feasible personalized treatment options for patients with these mutations. In addition to the trunk mutations, ESCC genomes exhibit a portion of subclonal mutations that must be considered in assessing responses to targeted therapies. A better understanding of the cancer evolution process of ESCC is essential to design rational approaches for cancer therapy and prevention.

In conclusion, we found that heterogeneity did not simply affect coding mutations in ESCC, genomic copy-number heterogeneity was also extensive across ESCC genomes. However, our analyses were limited in a smaller sample cohort. Further studies of larger patient cohorts from multiple restricted areas with high incidences of ESCC will be necessary to validate the CIN status and the relationship of CIN with clinical features, as well as to further refine the important therapeutic implications in guiding efforts to limit ESCC tumor diversity, evolution, and adaptation.

## MATERIALS AND METHODS

### Ethics statement

All patients have given their informed consent and all samples were obtained before treatment according to the guidelines of the local ethical committees (IRB of Shanxi Medical University & the Ethics Committee of Henan Cancer Hospital), with the approval letter of ethics committee of Shanxi Medical University (Approval No. 2009029) and Henan Cancer Hospital (Approval No. 2009xjs12).

### Data process and analysis

We employed a nonnegative matrix factorization (NMF) and model selection approach to extract mutational signatures from the WGS data of 17 ESCC tumors with GD [15]. The somatic CNVs of sample pairs were identified by algorithm based on Patchwork [33]. Segmentation results were then used for the subsequent analysis. GISTIC algorithm was used to infer recurrently high amplified genomic regions. Absolute copy number was estimated by ABSOLUTE v1.06 [9, 34, 35].

### Identification of GD events and GD timing

A published algorithm was used to identify the genome of tumors with GD [21]. Of note, 100,000 simulations were run for each sample. Genotype proportions reflecting copy numbers was used to represent each GD case. Only losses to two copies (AA, BB, and AB) were used, as these reflect losses either prior to (AA or BB) or after GD (AB). The A and B represent two parental alleles. Tumors with a higher proportion AB compared with AA/BB were classified as GD occurring before the majority of losses whereas those with AA/BB > AB were classified as GD occurring after the majority of losses.

### SCNAs timing relative to GD event

Gains or losses were determined by the modal absolute copy number of each chromosome arms. A modified version of a published method was used to classify the focal SCNAs with absolute copy number less than 6, via their timing relative to GD event [21, 36]. We considered deletions with an odd total copy number or odd minor copy number to likely occur after GD whereas other deletions were considered as events occurred prior to GD. Meantime, we called gains with an even total copy number change as occurring before GD and other gains with odd copy change as occurring after GD.

### Copy number altering score

A segment was defined as an abnormal variation if it had copy number gain or loss relative to the modal absolute copy number of chromosome arm. The copy number altering score represents the samples supporting percentage of abnormal variations for each gene region covered by segments. We considered those tumors harboring a segment that covered to the center of this gene region as the supporting samples of one gene. For each gene, denote the count of supporting samples is *k*_gene_ and sample size is *n*, we estimated the score by the following formula

$$Score_{\text{gene}}=\frac{k_{\text{gene}}}{n}.$$

In the same context (for example, gains), a gene has variated frequently that maybe contribute to solid tumors if the copy number altering score at least 0.4.

### Timing duplication events

To estimate the molecular time of duplication events, we adopted the approach described by Nik-Zainal *et al* (2012) and Greenman *et al* (2012). Firstly, utilizing Patchwork [33, 35], we separated the whole genome into hundreds of continuous segments and calculated the integer allelic specific copy number of each segment. Then, the multiplicity of somatic SNVs was estimated with ABSOLUTE, which could also distinguish clonal and subclonal SNVs. Reasoning mutations that arise early in tumorigenesis or that foster rapid outgrowth would tend to be clonal whereas late-arising alterations would more often be subclonal. Next, for each segments, the number of clonal mutations at same multiplicity value were summed. It is the fact that mutations with multiplicity = 1 usually occur after duplication while mutations with multiplicity > 1 tend to occur after duplication. To calculate the molecular time of arm level amplification, we merged the segments with same copy number mode on each arm and computed the fraction of each mode. The mode, whose fraction on each arm was larger than 0.75, was identified as the arm's mode. Finally, the fraction of mutation time of gains at arm level was estimated using the following set of equations. As noted above, *M_P_*is the number of mutations observed in each arm with multiplicity *P*.

Case1: Major=2, minor=0,

Proportional time of duplication:

$$T=M_{2}÷(M_{2}+M_{1}÷2).$$

Case2: Major=2, minor=1,

Proportional time of duplication

$$T=M_{2}÷(M_{2}+(M_{1}-M_{2})÷3).$$

Case3: Major=3, minor=0,

Proportional time of first duplication

T1=M÷3(M3+M2+(M1−M2)÷3);

Proportional time of second duplication

T2=(M+3M2)÷(M3+M2+(M1−M2)÷3).

Case4: Major=4, minor=0,

Proportional time of first duplication:

$$T_{1}=M_{4}÷(M_{4}+M_{2}÷2+M_{1}÷4);$$

Proportional time of second duplication:

$$T_{2}=(M_{4}+M_{2}÷2)÷(M_{4}+M_{2}÷2+M_{1}÷4).$$

Case5: Major=2, minor=2 (For genome doubled individuals),

Proportional time of duplication:

$$T=(M_{2}÷2)÷(M_{2}÷2+M_{1}÷4).$$

### qPCR copy number analysis

Copy number of *CDKN2A*, *FEN1*, *MTA2* and *ZBTB3* was assessed in frozen tumor samples and matched normal tissues as described [11]. Copy numbers were determined by real-time PCR with DNA binding dye SYBR Green I using three highly specific primer pairs that flanked three coding exons of the interesting gene, respectively. In a final volume of 25 μl, 20 ng DNA was amplified with SYBR Green PCR Master Mix (QIAGEN, Germany) in triplicate and RNase P (*RPPH1* gene; Life Technologies, 4403328) was used as a diploid control. Data was analyzed using the comparative (delta-Ct) Ct method. Inferred copy number of < 1 was considered to indicate a deletion and > 3 was considered as amplification.

### Immunohistochemistry and tissue microarray resource

Human esophageal squamous cell carcinoma tissue arrays that contain 36 atypical hyperplasia tissues, 72 ESCC tumors and matched adjacent normal esophagus tissues were prepared in our lab. Immunohistochemically staining of interesting proteins were performed as described [10, 11]. Briefly, sections were incubated with the antibody working dilution for 14 hours at 4°C, followed by detection using the PV8000 (Zhongshan) and DAB detection kit (Maixin), producing a dark brown precipitate. Slides were counterstained with hematoxylin. All images were captured at × 20. Expressions of proteins were analyzed using Aperio Nuclear v9 software. Statistical analyses were performed using Graghpad Prism 5.0. The staining intensity was scored as 0 (negative), 1 (weak), 2 (moderate), and 3 (strong). The immunoreactive score (IRS) was determined by the product of the extent score and the intensity score. Values of IRS ranging from 0 to 9, which were graded as follows: - (IRS 0), + (IRS 1-3), ++ (IRS 4-6), +++ (IRS 7-9). The median of IRS was chosen to define the cases.

## SUPPLEMENTARY MATERIALS FIGURES AND TABLES



## Abbreviations

BFB: breakage-fusion-bridge
CIN: Chromosomal instability
CNAs: copy-number alterations
COAD: colorectal carcinoma
ESCA: esophageal carcinoma
ESCC: esophageal squamous cell carcinoma
FEN1: Flap structure-specific Endonuclease 1
GD: genome doubling
HNSCC: head and neck squamous cell carcinoma
IHC: immunohischemistry
LOH: loss of heterozygosity
LIHC: liver hepatocellular carcinoma
NGD: non-genomedoubling
NLOH: neutral loss of heterozygosity
NMF: nonnegative matrix factorization
NSCLC: non-small cell lung cancer
PAAD: pancreatic adenocarcinoma
STAD: stomach adenocarcinoma
SCNA: somatic copy number alteration
SNV: single-nucleotide variations
TSGs: tumor suppressor genes
TCNAs: telomere-bounded copy number alterations
WGD: whole genome doubling
WGS: whole-genome sequencing
wGII: genome instability index
