# Supplementary material for: The macro-evolutionary events in esophageal squamous cell carcinoma
Source: Oncotarget. 2017 Nov 15;8(68):112770–82. doi: 10.18632/oncotarget.22625 (PMC5762549; doi:10.18632/oncotarget.22625)
Supplement: Supplementary file 1 [file oncotarget-08-112770-s001.pdf]

The macro-evolutionary events in esophageal squamous cell carcinoma

SUPPLEMENTARY MATERIALS

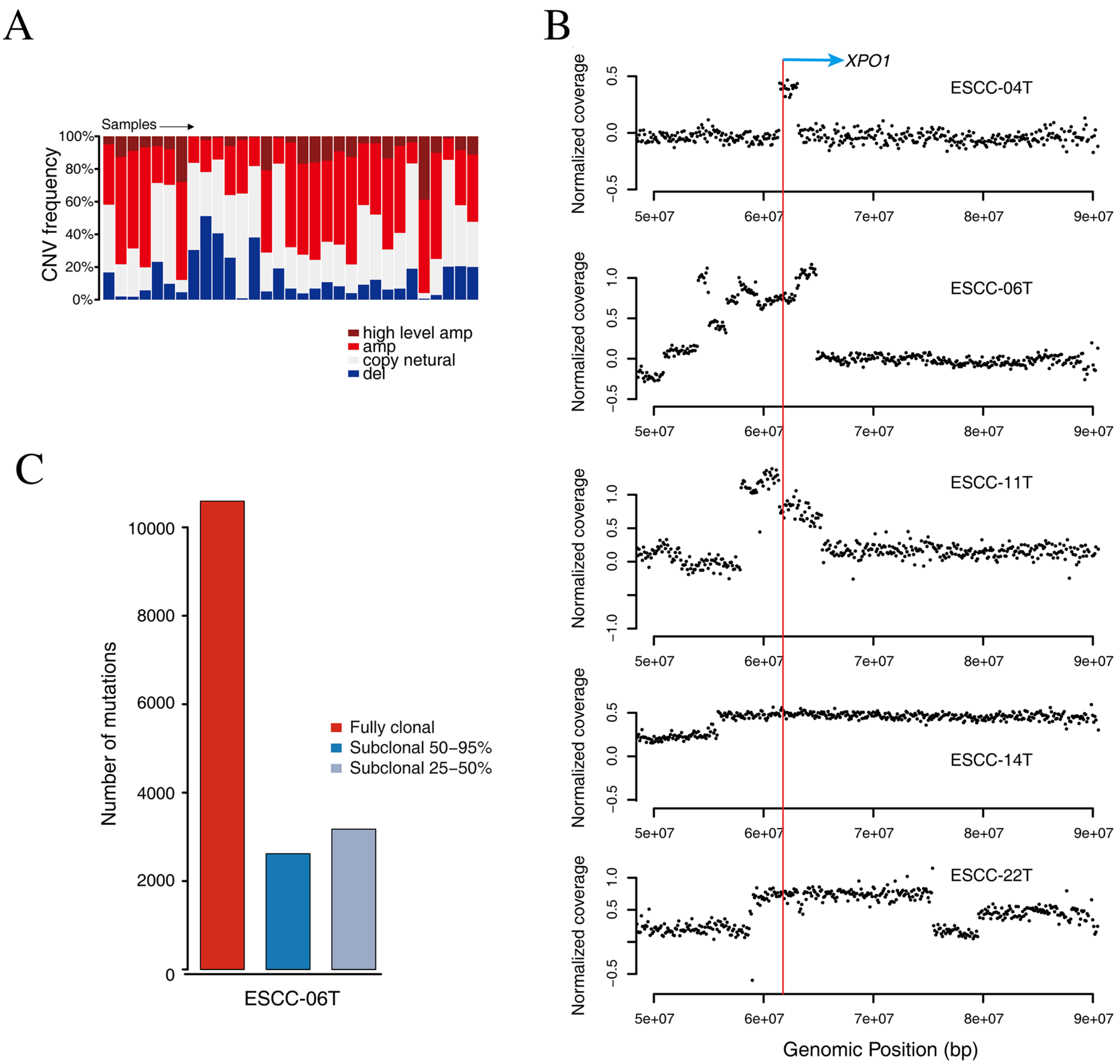

Supplementary Figure 1: (A) Bar graph shows the frequency of the deletions (blue), amplifications (red), and high-level amplifications (dark red) for 31 ESCCs. **(B)** Bar graph shows the number of fully clonal mutations (red bar), mutations in 50%–95% tumor cells (dark blue bar), and 25%–50% tumor cells (light blue bar) for ESCC-06T. **(C)** XPO1 is focally amplified in five ESCC genomes. The dot shows the log copy ratio along chromosome 2. The red line shows the chromosomal location of XPO1.

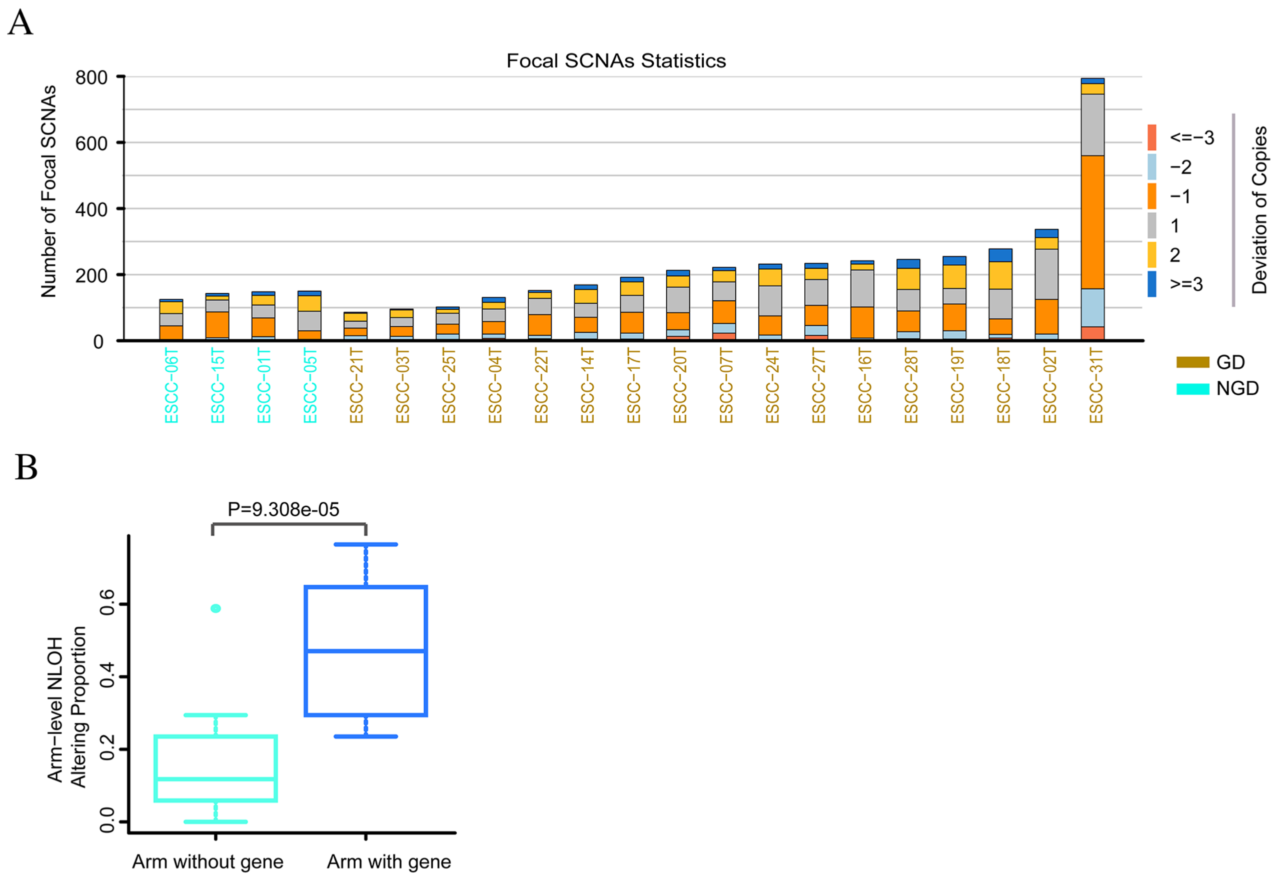

Supplementary Figure 2: (A) Deviation of copy number between tumors with GD and NGD. Different colors represent different deviation calculated by the following method: the absolute copy number of one segment - modal baseline of chromosome arm that involves this segment. Each column represents a sample. Horizontal axis represents those samples Listed from largest to smallest with number of focal SCNAs. Vertical axis represents the number of focal SCNAs. **(B)** Boxplot of arm-level NLOH altering proportion in chromosome arms with or without tumor suppressor gene. Wilcoxon rank sum test was used to compare the difference between the two groups.

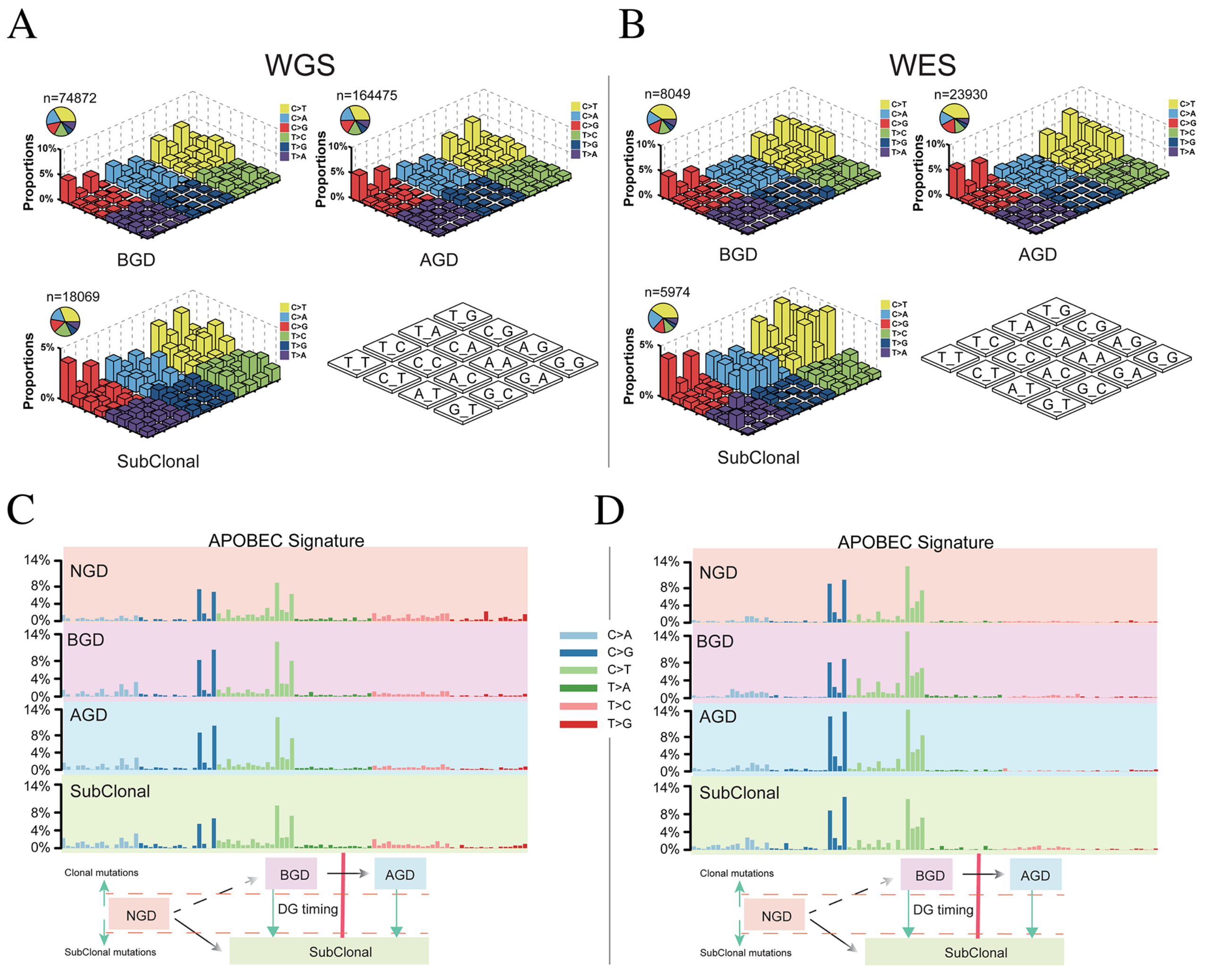

Supplementary Figure 3: In the panel, this shown on the left is WGS data from 17 samples with GD, and on the right is WES data from 96 samples with GD. From the top, mutation spectrums of BGD (before genome doubling) set, AGD (after genome doubling) set and subclonal (subclonal mutations) set are shown in **(A)** and **(B)**. Then 2 APOBEC signature of each groups and the simple evolutionary diagram between the four groups are exhibited in **(C)** and **(D)**, of which the solid line arrows represent the evolutionary process and the gray arrows represent events that likely to occur later.

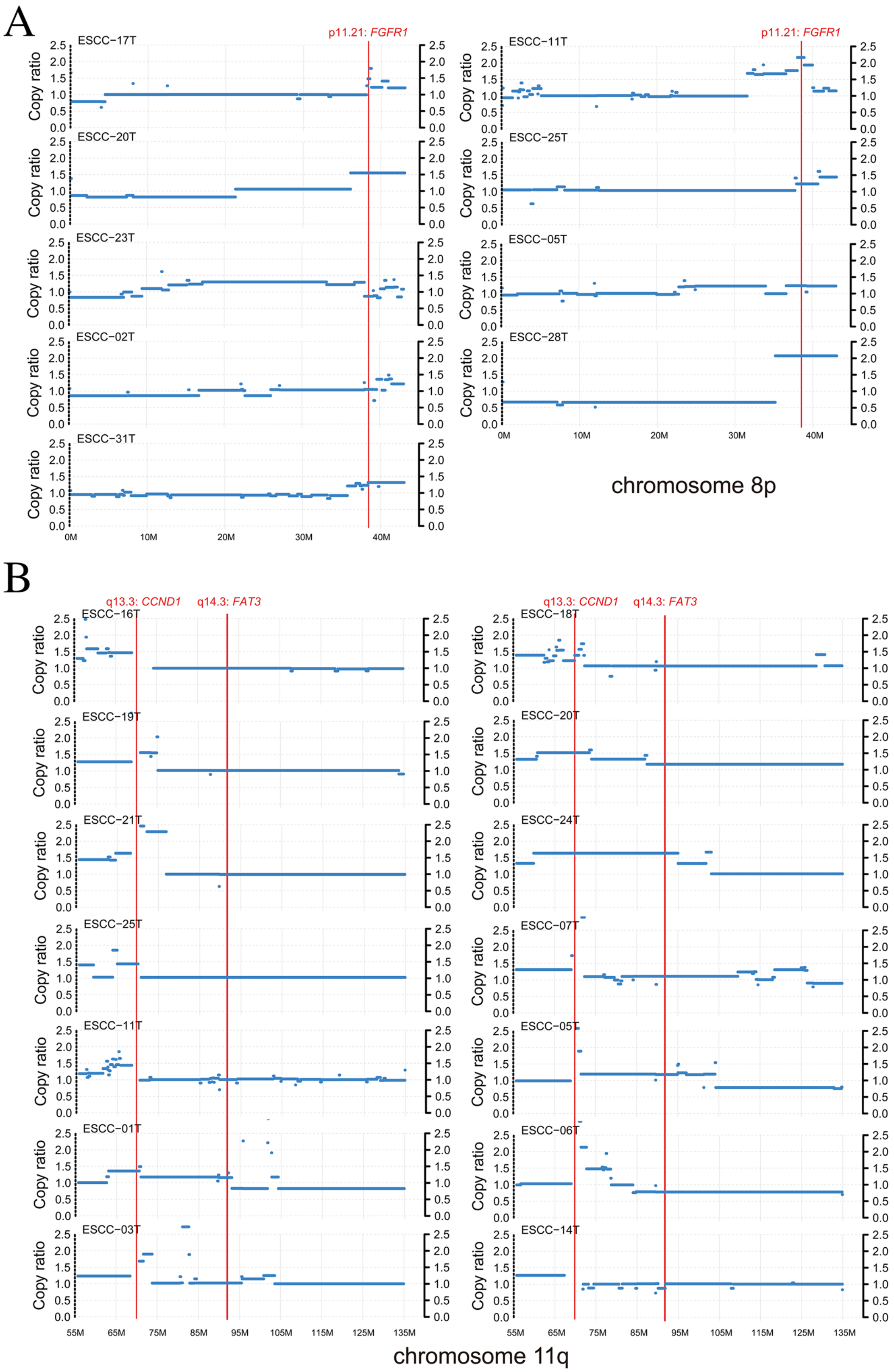

Supplementary Figure 4: Copy number changes involving genes FGFR1, CCND1 and FAT3. (A) FGFR1 amplification was found in 9 cases. **(B)** CCND1 amplification accompany with telomere-bounded FAT3 deletion in the majority of CCND1-amplified ESCCs.

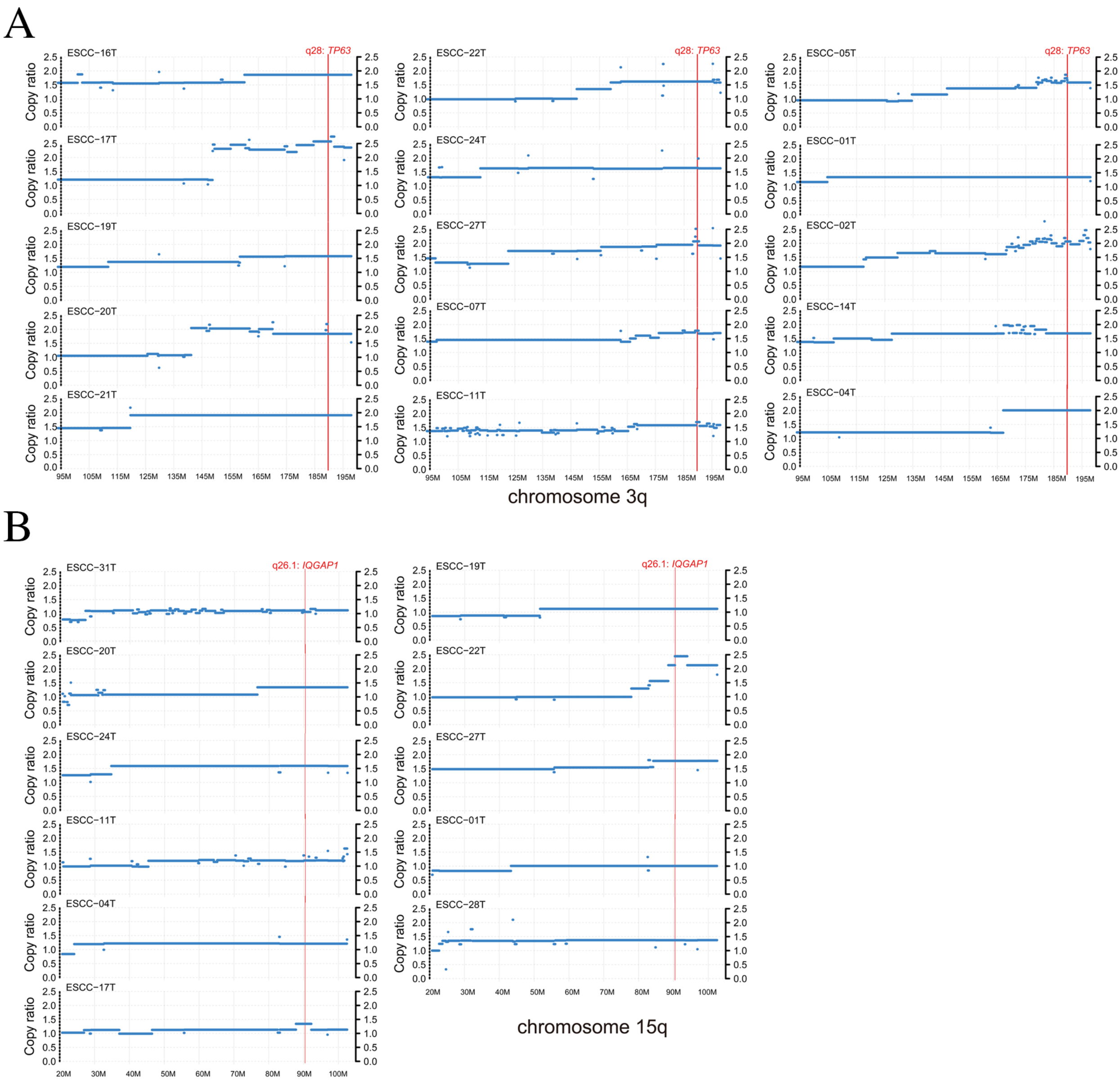

Supplementary Figure 5: Telomere-bounded amplification in chromosome arm 3q and 15q respectively. **(A)** Gene TP63 (red line location) demonstrates both telomere-bounded and focal amplification. **(B)** Gene IQGAP harbors telomere-bounded or focal amplifications in chromosome 15q.

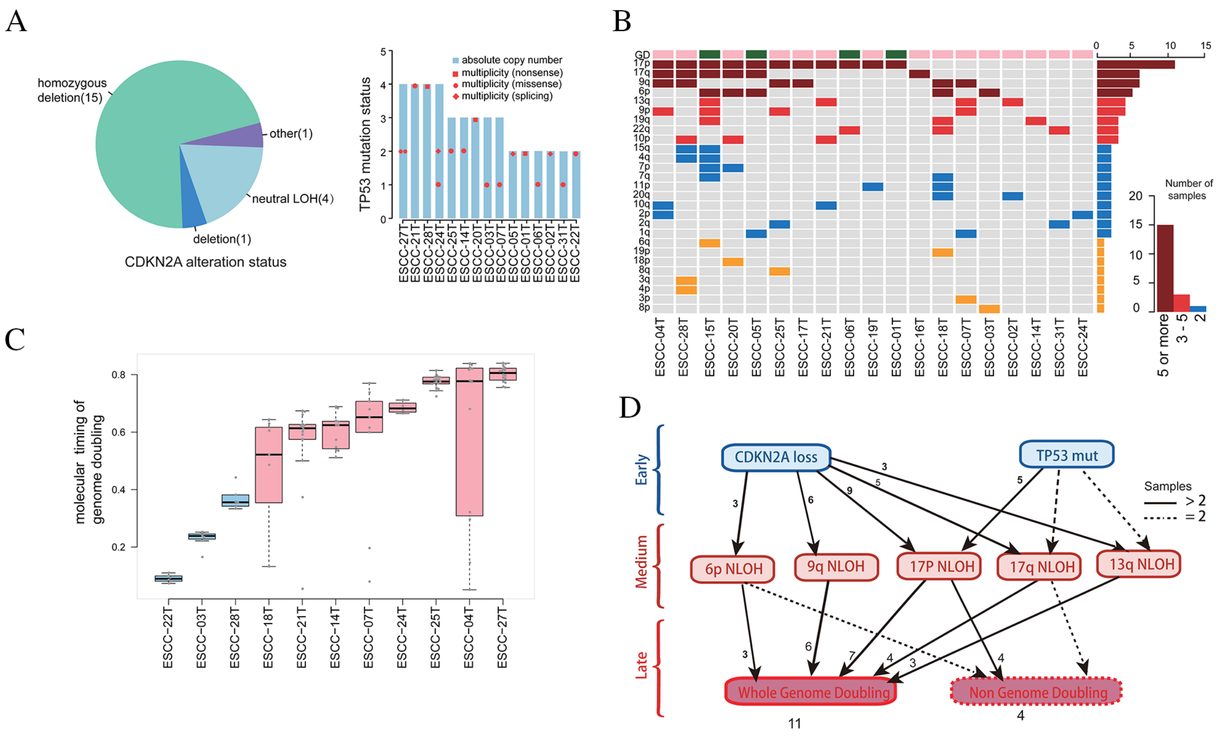

Supplementary Figure 6: Matched patterns at different evolutionary steps. (A) The proportion and number of mutated CDKN2A and TP53. **(B)** Distribution of intrinsic NLOH across 19 samples. **(C)** Estimated time of genome doubling event in 11 samples. Blue and red box represents the early and late genome doubling events. **(D)** Inferred evolutionary history of ESCC. Edges are drawn when the connection are found in at least two samples. Early, Medium and late are inferred according to their molecular time.

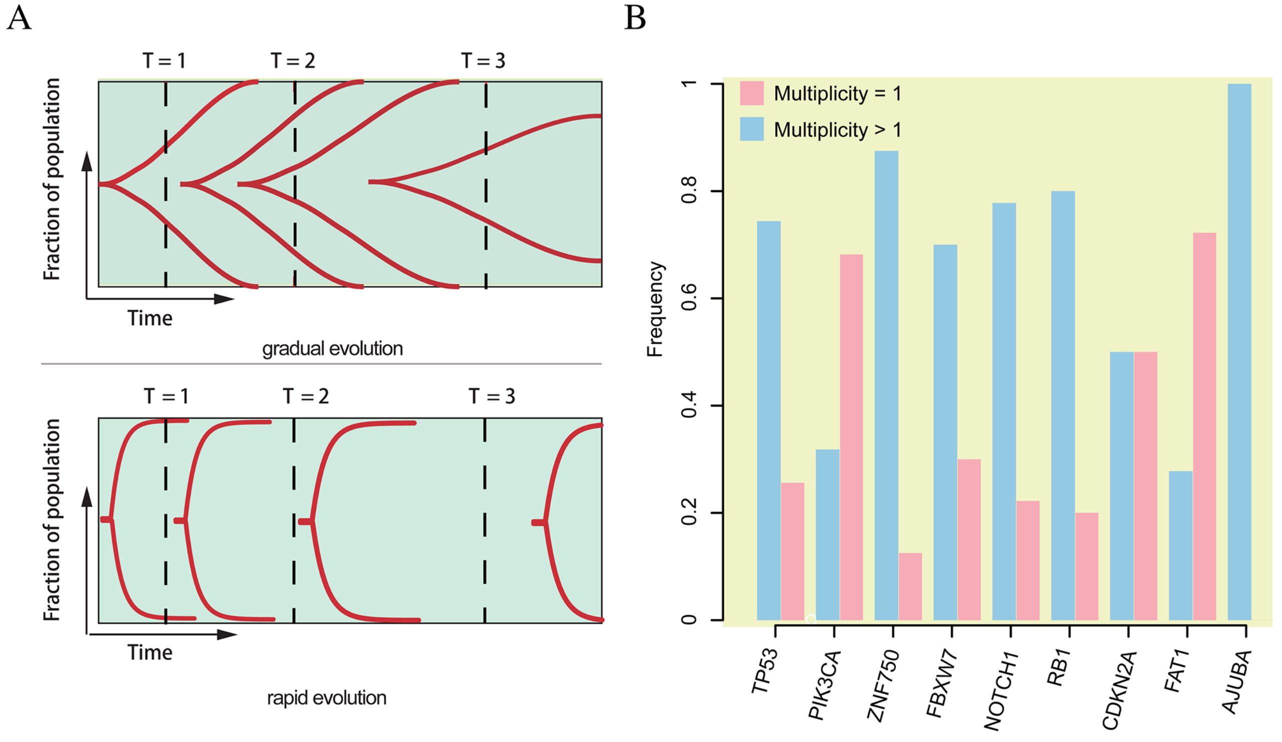

Supplementary Figure 7: (A) The top model represents gradual evolution and the bottom represents rapid evolution. **(B)** Each bar shows the frequency of driver gene in different status.

Supplementary Table 1: Genomic info of 21 ESCC patients with absolute copy number available.

See Supplementary File 1

Supplementary Table 2: Recurrent cancer gene status of 21 ESCC patients.

See Supplementary File 2
